# Effectiveness of the primary Bacillus Calmette-Guérin vaccine against the risk of *Mycobacterium tuberculosis* infection and tuberculosis disease: a meta-analysis of individual participant data

**DOI:** 10.1016/j.lanmic.2024.100961

**Published:** 2024-12-19

**Authors:** Puck T Pelzer, Logan Stuck, Leonardo Martinez, Alexandra S Richards, Carlos Acuña-Villaorduña, Naomi E Aronson, Maryline Bonnet, Anna C Carvalho, Pei-Chun Chan, Li-Min Huang, Chi-Tai Fang, Gavin Churchyard, Helena del Corral-Londoño, Manjula Datta, Marcos A Espinal, Katherine Fielding, Andrew J Fiore-Gartland, Alberto Garcia-Basteiro, Willem Hanekom, Mark Hatherill, Phillip C Hill, Helena Huerga, Edward C Jones-López, Afranio Kritski, Anna M Mandalakas, Punam Mangtani, Eduardo Martins Netto, Harriet Mayanja, Rufaida Mazahir, Megan Murray, Molebogeng Rangaka, Thomas Scriba, Jitendra Singh, Sarman Singh, Catherine M Stein, Johan Vekemans, Lilly M Verhagen, Julian A Villalba, Anne Wajja, Basilea Watson, Richard G White, Frank G J Cobelens

**Affiliations:** Amsterdam University Medical Centres, Amsterdam, Netherlands (P T Pelzer MSc, Prof F G J Cobelens PhD, L Stuck PhD); KNCV Tuberculosis Foundation, The Hague, Netherlands (P T Pelzer); London School of Hygiene & Tropical Medicine, London, UK (P T Pelzer, A S Richards PhD, Prof R G White PhD, Prof K Fielding PhD, Prof P Mangtani FFPHM); Department of Global Health and Amsterdam Institute for Global Health and Development, Amsterdam, Netherlands (P T Pelzer, Prof F G J Cobelens, L Stuck); Department of Epidemiology, School of Public Health, Boston University, Boston, MA, USA (L Martinez PhD); Boston University Medical Center, Boston, MA, USA (C Acuña-Villaorduña MD); Uniformed Services University of the Health Sciences, Bethesda, MD, USA (Prof N E Aronson MD); TransVIHMI, University of Montpellier, IRD, INSERM, Montpellier, France (M Bonnet PhD); Laboratório de Inovações em Terapias, Ensino e Bioprodutos, Instituto Oswaldo Cruz, Fundação Oswaldo Cruz, Rio de Janeiro, Brazil (A C Carvalho PhD); Division of Chronic Infectious Disease, Taiwan Centers for Disease Control, Taipei, Taiwan (Prof P-C Chan PhD); Department of Pediatrics, National Taiwan University Hospital and National Taiwan University College of Medicine, Taipei, Taiwan (Prof P-C Chan, Prof C-T Fang PhD); Institute of Epidemiology and Preventive Medicine, College of Public Health, National Taiwan University, Taipei, Taiwan (Prof L-M Huang PhD, Prof P-C Chan); Aurum Institute, Johannesburg, South Africa (Prof G Churchyard PhD); School of Public Health, University of the Witwatersrand, Johannesburg, South Africa (Prof G Churchyard); Department of Medicine, Vanderbilt University, Nashville, TN, USA (Prof G Churchyard); Universidad de Antioquia, National School of Public Health, Medellin, Colombia (H del Corral-Londoño PhD); A Society for Primary Health Care Intervention, Research and Education, Chennai, India (M Datta MSc); Programa Acadêmico de Tuberculose, Faculdade de Medicina, Universidade Federal do Rio de Janeiro, Rio de Janeiro, Brazil (A Kritski MSc); National Centre for Research on Maternal and Child Health, Santo Domingo, Dominican Republic (M A Espinal MD); Infectious Disease Epidemiology Department, Epicentre, Paris, France (Prof K Fielding, Prof P Mangtani); Fred Hutchinson Cancer Center, Seattle, WA, USA (A J Fiore-Gartland PhD); Barcelona Institute for Global Health, Barcelona, Spain (A Garcia-Basteiro PhD); Africa Health Research Institute, Durban, South Africa (Prof W Hanekom PhD); South African Tuberculosis Vaccine Initiative, Institute of Infectious Disease and Molecular Medicine, Division of Immunology, Department of Pathology, University of Cape Town, Cape Town, South Africa (Prof M Hatherill MD); Centre for International Health, University of Otago, Dunedin, New Zealand (Prof P C Hill MD); Field Epidemiology Department, Epicentre, Paris, France (H Huerga PhD); Division of Infectious Diseases, Department of Medicine, Keck School of Medicine of USC, University of Southern California, Los Angeles, CA, USA (E C Jones-López MSc); Baylor College of Medicine, Houston, TX, USA (Prof A M Mandalakas MD PhD); Research Center Borstel, Sülfeld, Germany (Prof A M Mandalakas); Faculdade de Medicina da Bahia, Salvador, Brazil (Prof E Martins Netto MD PhD); Hospital Universitário Professor Edgard Santos, Salvador, Brazil (Prof E Martins Netto); Universidade Federal da Bahia, Salvador, Brazil (Prof E Martins Netto); Makerere University Infectious Diseases Institute, Kampala, Uganda (Prof H Mayanja MBChB MMed MSc); Nehru Medical College, Aligarh Muslim University, Aligarh, Uttar Pradesh, India (R Mazahir MD PhD); Department of Global Health and Social Medicine, Harvard Medical School, Boston, MA, USA (Prof M Murray PhD); Institute for Global Health and MRC Clinical Trials Unit, University College London, London, UK (Prof M Rangaka PhD); South African Tuberculosis Vaccine Initiative, Institute of Infectious Disease and Molecular Medicine, Division of Immunology, Department of Pathology, University of Cape Town, Cape Town, South Africa (Prof T Scriba PhD); Department of Translational Medicine, All India Institute of Medical Sciences Bhopal, Madhya Pradesh, India (J Singh PhD); Department of Laboratory Medicine, All India Institute of Medical Sciences, New Delhi, India (J Singh); All India Institute of Medical Sciences, New Delhi, India (Prof S Singh MD); All India Institute of Medical Sciences, Bhopal, India (Prof S Singh); Aaarupadai Veedu Medical College, Pondicherry, India (Prof S Singh); Department of Population and Quantitative Health Sciences, and Division of Infectious Diseases and HIV Medicine, Department of Medicine, Case Western Reserve University, Cleveland, OH, USA (C M Stein PhD); IAVI, New York, NY, USA (J Vekemans PhD MD); Department of Pediatric Infectious Diseases and Immunology, Radboud Community for Infectious Diseases, Amalia Children’s Hospital, Radboud University Medical Center, Nijmegen, Netherlands (L M Verhagen PhD MD); Department of Laboratory Medicine, Laboratory of Medical Immunology, Radboud University Medical Center, Nijmegen, Netherlands (L M Verhagen); Department of Paediatrics and Child Health, Faculty of Medicine and Health Sciences, Stellenbosch University, Cape Town, South Africa (L M Verhagen); Laboratorio de Tuberculosis, Instituto de Biomedicina, Universidad Central de Venezuela, Caracas, Venezuela (J A Villalba PhD MD); Department of Pathology and Laboratory Medicine, Emory University School of Medicine, Atlanta, GA, USA (J A Villalba); Makerere University Infectious Diseases Institute, Kampala, Uganda (A Wajja MBChB); ICMR-National Institute for Research in Tuberculosis, Chennai, India (B Watson MSc)

## Abstract

**Background:**

Tuberculosis vaccine trials using disease as the primary endpoint are large, time consuming, and expensive. An earlier immunological measure of the protection against disease would accelerate tuberculosis vaccine development. We aimed to assess whether the effectiveness of the Bacillus Calmette-Guérin (BCG) vaccine for prevention of *Mycobacterium tuberculosis* infection was consistent with that for prevention of tuberculosis disease.

**Methods:**

We conducted an individual participant data (IPD) meta-analysis on experimental and observational longitudinal studies before April 6, 2018, identified through systematic reviews, known to us through expert knowledge in the field, reporting on BCG vaccination status, *M tuberculosis* infection test (QuantiFERON IFN-γ release assay [IGRA] and tuberculin skin test [TST]), and tuberculosis incidence. Cohort studies were included only for countries with a mandatory neonatal BCG vaccination policy. Exclusion criteria were previous or current tuberculosis disease, HIV infection, tuberculosis preventive treatment usage, and for household contacts, a positive baseline IGRA or TST test and young children aged 0–2 years; for randomised controlled trials, TST results within 2 years after random assignation were excluded. We contacted the investigators of the identified studies to provide IPD. We compared the protective efficacy of the BCG vaccine against *M tuberculosis* infection with that against tuberculosis disease using mixed-effects, multivariable proportional hazards modelling, by study type, *M tuberculosis* infection test (IGRA and TST), cutoff for defining test positivity, age, sex, and latitude.

**Findings:**

We identified 79 studies eligible for full screening and of these, IPD datasets from 14 studies were included in our analysis: 11 household contact studies (29 147 participants), two adolescent cohort studies (11 368 participants), and one randomised controlled trial (2963 participants). Among 28 188 participants we found no protection by the BCG vaccine against TST conversion regardless of cutoff in any type of study. Among 1491 household contacts, but not among 5644 adolescents, the BCG vaccine protected against QuantiFERON conversion at the primary cutoff of 0⋅7 IU/mL or more with the adjusted hazard ratio (0⋅65, 95% CI 0⋅51–0⋅82) being consistent with that for protection against disease (0⋅68, 0⋅18–2⋅59). Protection against QuantiFERON conversion at cutoff of 0⋅35 IU/mL or more (0⋅64, 0⋅51–0⋅81) was similar.

**Interpretation:**

Protection from the BCG vaccination against *M tuberculosis* infection, measured as QuantiFERON conversion, is inconsistent across different groups. Among groups with recent household exposure, QuantiFERON conversion is consistent with protection against disease and could be evaluated as a proxy for disease in tuberculosis vaccine trials. We found that TST lacks value for prevention in phase 2b proof-of-concept trials.

**Funding:**

Bill & Melinda Gates Foundation.

## Introduction

Tuberculosis remains a major health problem with 1⋅3 million deaths in 2022.^1^ The Bacillus Calmette-Guérin (BCG) vaccine, the only currently available tuberculosis vaccine, has been in use since 1921 and is recommended for neonates in tuberculosis-prevalent countries in whom it consistently prevents miliary disease and tuberculosis meningitis.^2^ However, the BCG vaccine offers variable protection for adults who are more likely to transmit *Mycobacterium tuberculosis*, the causative agent of tuberculosis.^3,4^ Efforts to address this limitation have led to new tuberculosis vaccine candidates, with at least 14 currently in the pipeline.^5^ Despite progress in research, the lack of adequate funding and political support remains a major obstacle to their development.

The endpoint for regulatory phase 3 trials of new vaccines developed for preventing tuberculosis in adolescents and adults is prevention of disease, which requires large sample sizes with prolonged follow-up.^4,5^ A major impediment to acceleration of clinical development of new tuberculosis vaccine candidates is the absence of validated proxy effectiveness endpoints that could be used to establish proof-of-principle in phase 2b trials to guide decisions about moving a candidate forward to phase 3 trials.^6^ One proposed proxy endpoint is prevention of *M tuberculosis* infection.^7^ Several studies have suggested that the BCG vaccine provides protection against *M tuberculosis* infection.^4,8–12^ Traditional diagnostics for *M tuberculosis* infection include IFN-γ release assays (IGRA) and the tuberculin skin test (TST). However, both tests are unable to determine recent infection, and the challenge lies in discerning whether infection serves as a biomarker for disease or merely represents a milestone in the natural history of tuberculosis.^13^

For either diagnostic method, we do not know how well prevention of *M tuberculosis* infection predicts protection for prevention of disease. In case *M tuberculosis* infections prevented by vaccination are primarily those that do not progress to tuberculosis disease, a vaccine candidate showing substantial prevention of *Mtuberculosis* infection in a phase 2b study would fail to show prevention of disease in a subsequent phase 3 trial. Conversely, the protection afforded by a vaccine might be mainly or entirely through preventing disease among those already infected. In such a case, a phase 2b trial might show no prevention of *M tuberculosis* infection effect, and the vaccine candidate might incorrectly be discarded for a subsequent phase 3 trial.

Previous systematic reviews have explored the effectiveness of the BCG vaccine in preventing infection and disease independently^2,6–11^ or have assessed the predictive value of tests for infection without considering vaccination status.^12,13^ We aimed to perform an individual participant data (IPD) meta-analysis of observational studies and randomised trials to understand to what extent prevention afforded by primary BCG vaccination of *M tuberculosis* infection as determined by TST or IGRA is consistent with prevention of incident tuberculosis disease, overall and by specific subgroups.

## Methods

### Search strategy and selection criteria

In this IPD meta-analysis we screened published systematic reviews and meta-analyses of randomised controlled trials (RCTs) and observational studies for inclusion (appendix pp 4–7).^4,10,14,15^ This IPD meta-analysis builds upon four systematic reviews: one from 2013, another from 2014, and two from 2020. These reviews form the foundation of our current analysis, offering a comprehensive overview of available data from the early 1900s to 2019. Cohort studies conducted to estimate *M tuberculosis* infection and disease incidence in preparation of tuberculosis vaccine trials were suggested by an expert (FGJC) and included in the screening.^16–18^

We included experimental and observational studies with longitudinal follow-up that reported BCG vaccination status, *M tuberculosis* infection test (IGRA or TST, or both), and tuberculosis disease incidence. Cohort studies were included only for countries with a mandatory neonatal BCG vaccination policy. We included prospective and retrospective cohort studies, as well as clinical trials, where participants were required to have a negative TST or IGRA before BCG vaccination or placebo. Studies needed at least a 2-year interval between BCG vaccination and subsequent TST or IGRA testing, along with ascertainment of tuberculosis disease. We excluded studies that did not have information on BCG status, IGRA or TST results, and tuberculosis disease outcomes, as well as those involving tuberculosis preventive treatments. We contacted the investigators of the identified studies to provide individual-level data.

Among the cohort datasets, we assumed absence of *M tuberculosis* infection prior to BCG vaccination. We conducted data validation through comparison of demographic, exposure, and outcome data with those reported in the original publications. Any inconsistencies were addressed through discussions with investigators.

We assessed three types of study design: RCTs, household contact studies, and adolescent cohort studies (ACSs). We applied standardised exclusion criteria on all datasets: participants with previous or current tuberculosis disease (within 60 days), HIV infection, baseline positive IGRA or TST tests among household contacts, and children aged 0–2 years were excluded. For RCTs, studies reporting TST results within 2 years after random assignment were also excluded (appendix p 9). One author (PTP) evaluated the eligibility of studies, the screening process, and the data extraction that was done based on previous systematic reviews. Previous heterogeneity assessments of the included studies found low heterogeneity.^3^ We excluded observations with a time interval between BCG vaccination and the first test for *M tuberculosis* infection of less than 2 years. From the analyses we excluded RCT participants who had a positive *M tuberculosis* infection test at vaccination and household contact participants who had a baseline positive infection test (hereafter referred to as baseline positives).

BCG vaccination status was based on presence of a scar or vaccination medical records. We defined *M tuberculosis* infection as a positive TST of 15 mm or more or 10 mm or more induration and a QuantiFERON-TB Gold In-Tube (QFT; the only IGRA performed in the studies included in the IPD meta-analysis) testwith IFN-γ levels for tuberculosis Ag-nil of 0⋅7 IU/mL or more or 0⋅35 IU/mL or more that followed a test result below these cutoffs.^8,9,19–21^ Sustained conversion was defined as two subsequent positive QFT results with at least a 6-month interval between them.^19^ Various methods have been used in the past to deal with the cross-reaction problem of the BCG vaccine (appendix pp 4–7); in this study we aimed to increase specificity by choosing higher cutoffs.^8,9^ In all analyses, tuberculosis disease was defined as in the original studies irrespective of bacteriological confirmation due to absence of available data.

The IPD meta-analysis followed PRISMA guidelines.

### Data analysis

We conducted a one-stage IPD meta-analysis, combining data from multiple studies into a single dataset for analysis, accounting for the clustering of participants within studies. We performed three main analyses: by the method of measuring infection (ie, TST, IGRA, or sustained IGRA). Within each analysis, we examined various cutoffs. We used Cox proportional hazards regression models, a longitudinal analytical method that analyses the association between the BCG vaccine and *M tuberculosis* infection and tuberculosis disease, while accounting for differences between studies and between individuals.

We constructed a two-level mixed-effects Cox proportional hazards model, in which the first level corresponded to the specific study and the second level represented individuals. We calculated, in separate models, the hazard ratios (HRs) by BCG vaccination status for the infection outcome among all participants, for the disease outcome among all participants, and for the disease outcome among those participants who had a positive infection test, both crude and adjusted for age and sex.

For each analysis the outcomes were summarised by comparing, among all study participants, the age and sex adjusted HR (aHR) for disease with the aHR for infection as determined by TST or IGRA conversion and classified pragmatically: (1) protection against conversion agrees with protection against disease (aHR for conversion <1 with 95% CIs not including 1, and within an arbitrary 0⋅1 of the aHR for disease); (2) protection against conversion underestimates protection against disease (aHR for conversion <1 with 95% CIs not including 1, but aHR >0⋅1 larger than aHR for disease); (3) protection against conversion over-estimates protection against disease (direction of the HR is similar but the aHR for conversion is >0⋅1 smaller than aHR for disease); and (4) no protection against conversion (aHR for conversion >1, or <1 with 95% CI including 1). We considered the strength of statistical significance regarding protection against TST or IGRA conversion. Due to the small number of disease events, we did not prioritise statistical significance for protection against disease. If prevention of infection agreed with prevention of disease and overestimation occurred, it was interpreted as prevention of *M tuberculosis* infection is consistent with prevention of disease.

We used Kaplan–Meier curves to examine the effects of BCG vaccination over follow-up time.

We presented results by age, sex, and geographical location (appendix pp 4–7). We did sensitivity analyses to address potential biases, exploring test cutoff, multivariable analysis using Poisson regression to account for different study designs, BCG vaccination scar identification, proportion of the study population that were BCG vaccinated and, for the randomised trial data, duration of follow-up beyond 15 years.

Furthermore, to better understand the observed patterns of agreement between prevention of disease and IGRA conversion, we explored the relation to prevention of disease effects for different segments of IGRA conversion values, defining these segments as (1) sustained conversions only, (2) conversions at 0⋅7 IU/mL but not sustained, and (3) conversions at 0⋅35 IU/mL but below 0⋅7 IU/mL and not sustained.

### Role of the funding source

The funder of the study advised on study design. The funder of the study had no role in data collection, data analysis, data interpretation, or writing of the report.

## Results

**Figure 1:**
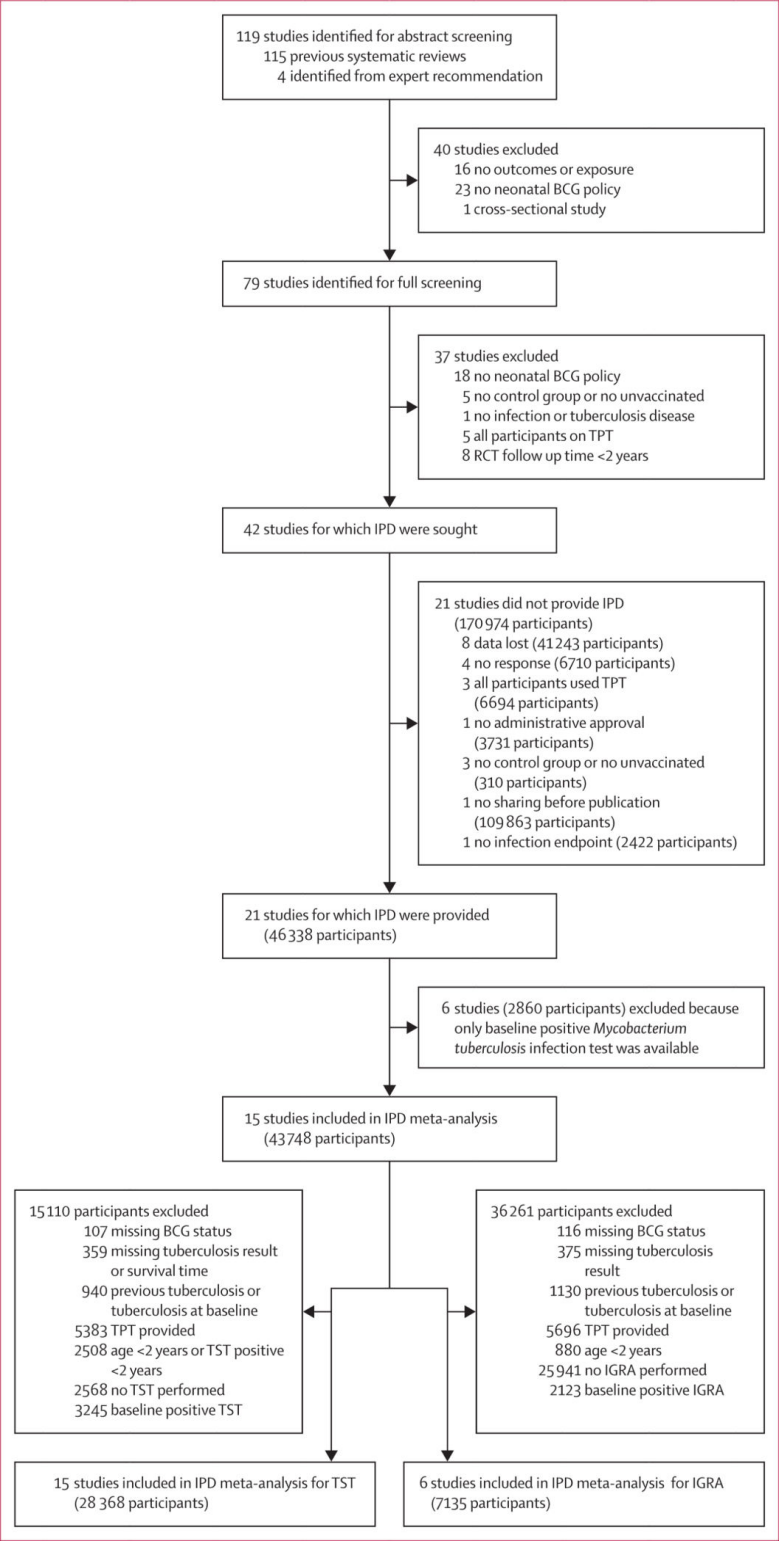
Study and participant selection BCG=Bacillus Calmette-Guérin. IGRA=IFN-γ release assay. IPD=individual patient data. RCT=randomised controlled trial. TPT=tuberculosis preventive treatment. TST=tuberculin skin test.

We identified 119 studies, of which 79 were eligible for full screening (figure 1). After full screening, we sought IPD for 42 studies. Of these, IPD could not be retrieved for 21 studies. For the remaining 21 studies data were provided, and six household contact studies were excluded because only one baseline test for *M tuberculosis* infection had been conducted. A study initially reported as an RCT in the review was excluded from our analysis after the principal investigator clarified it did not have a randomised design, resulting in its reclassification as an observational study with insufficient sample size.

14 datasets were included in our analysis (table). The full demographic characteristics of the original 29 147 household contacts (11 studies^22–32^; 15 423 [52⋅9%] female, 13 722 [47⋅1%] male, and two [0⋅0%] missing), 11 368 ACS participants (two studies^17,18^; 5872 [51⋅7%] female and 5496 [48⋅3%] male), and 2963 RCT dataset participants (one study^33^; 1515 [51⋅1%] female and 1448 [48⋅9%] male) are in the appendix (pp 13–20). IPD exclusion criteria were applied for each sub-analysis (appendix pp 10–12; showing the exclusion process for TST at 15 mm cutoff and IGRA at cutoff 0⋅7 IU/mL by study type). All ACS, household contact, and RCT datasets had TST data; seven studies had both tests,^18,24–26,28,31,32^ of which six^18,24,26,28,31,32^ were eligible for the longitudinal IGRA analysis.

**Figure 2:**
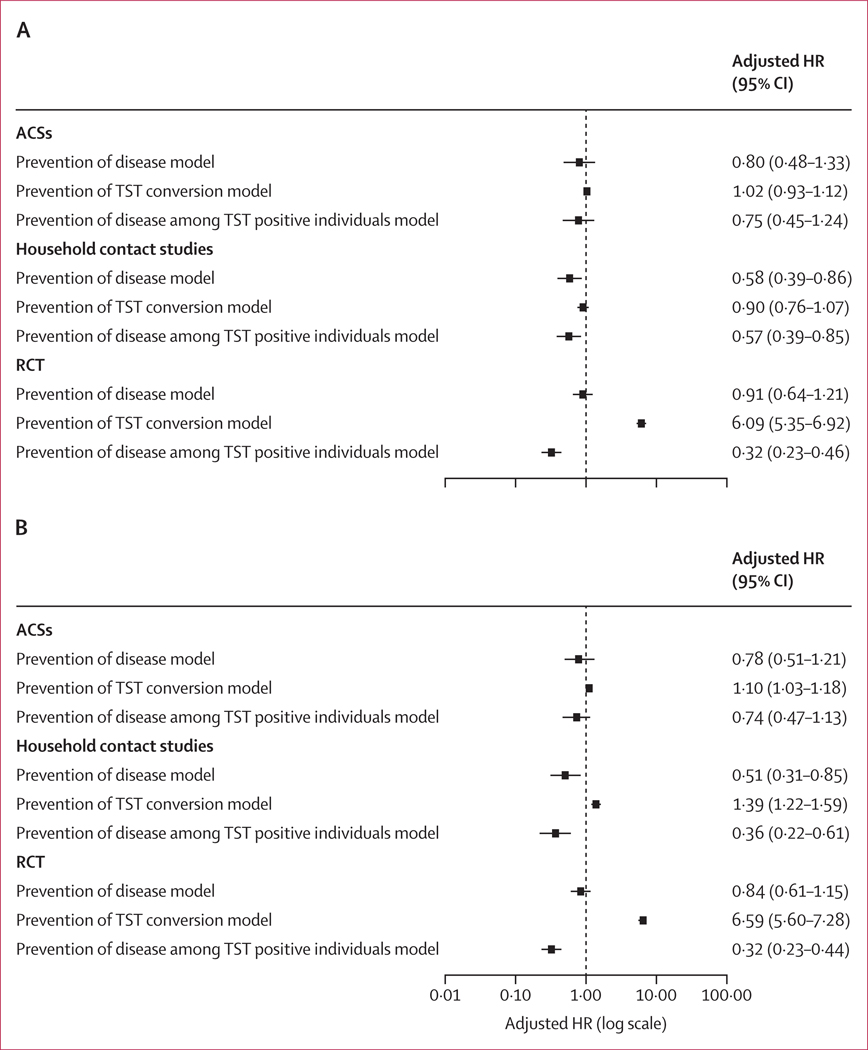
Adjusted models for ACSs, household contact studies, and an RCT, using a single conversion of 15 mm (A) and 10mm (B) cutoffs to determine TST positivity TST was assessed at any timepoint for ACSs, after baseline for household contact studies, and after 2 years for the RCT. Tuberculosis disease was assessed after 60 days of follow-up. Adjusted models showing Cox proportional mixed-effects model BCG and respective outcome adjusted for study, age, and sex. ACS=adolescent cohort study. BCG=Bacillus Calmette-Guérin. HR=hazard ratio. RCT=randomised controlled trial. TST=tuberculin skin test.

For the analyses based on TST at cutoff 15 mm, after applying exclusion criteria, the number of contributing participants was 10 350 for ACSs, 15 875 for the household contact studies, and 1963 for the RCT (appendix pp 21–23). In these analyses, there was no association between BCG vaccination status and TST conversion in the ACSs (aHR 1⋅02, 95% CI 0⋅93–1⋅12) or in the household contact studies (0⋅90, 0⋅76–1⋅07). In the RCTs, BCG vaccination showed an inverse effect (6⋅09, 5⋅35–6⋅92; figure 2A). These HRs were far from agreement with those for protection against disease that were 0⋅80 (0⋅48−1⋅33) for ACSs, 0⋅58 (0⋅39−0⋅86) for the household contact studies, and 0⋅91 (0⋅64−1⋅21) in the RCT, of which only that in the household contact studies was significantly smaller than 1 (0⋅58, 0⋅39–0⋅86).

When applying the 10 mm cutoff to determine TST positivity (appendix pp 24–26), in all study types the aHR was inverted: 1⋅10 (95% CI 1⋅03–1⋅18) in the ACSs, 1⋅39 (1⋅22–1⋅59) in the household contact studies, and 6⋅59 (5⋅60–7⋅28) in the RCT (figure 2B).

The absence of association between BCG vaccination and TST positivity was consistent across most studies except for evidence of some protection in two household contact studies^23,31^ (appendix pp 27–28, 30).

**Figure 3:**
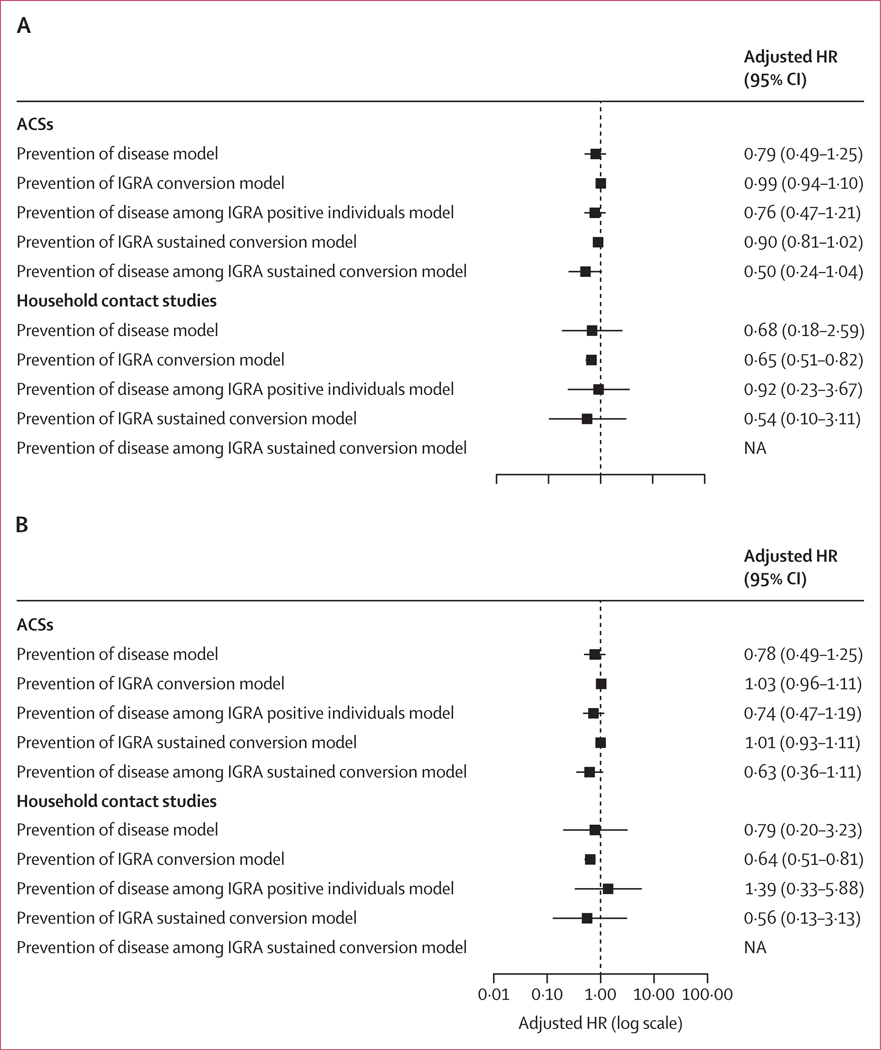
Adjusted models for ACSs and household contact studies using 0⋅7 IU/mL (A) and 0⋅35 IU/mL (B) cutoffs to determine IGRA positivity IGRA was assessed at any timepoint for ACSs and after baseline for household contact studies. Tuberculosis disease was assessed after 60 days of follow-up. Adjusted models showing Cox proportional mixed-effects model BCG and respective outcome adjusted for study, age, and sex. ACS=adolescent cohort study. BCG=Bacillus Calmette-Guérin. HR=hazard ratio. IGRA=INF-γ release assay. NA=not applicable.

One ACS,^18^ five household contact,^24,26,28,31,32^ and no RCTs had IGRA data. For the analyses based on an IGRA at cutoff of 0⋅7 IU/mL, the ACSs contributed 5644 participants, and after excluding participants with a positive baseline test at this cutoff the household contact contributed 1491 participants (appendix pp 29, 31). In the ACSs, there was no association between BCG vaccination and IGRA conversion (aHR 0⋅99, 95% CI 0⋅94–1⋅00), tuberculosis disease overall (0⋅79, 0⋅49–1⋅25), or tuberculosis disease among IGRA positive individuals (0⋅76, 0⋅47–1⋅21; figure 3A; n=5644). In the household contact dataset (n=1491), there was a reduced hazard of IGRA conversion among individuals vaccinated with BCG (0⋅65, 0⋅51–0⋅82). The point estimate of the hazard of disease (0⋅68, 0⋅18–2⋅59) was consistent with this reduced hazard, and no association was observed between BCG vaccination and disease among individuals who were IGRA positive (0⋅92, 0⋅23–3⋅67; figure 3A). In the ACSs, using an IGRA cutoff of 0⋅35 IU/mL, there was no association with BCG vaccination and IGRA conversion (1⋅03, 0⋅96–1⋅11; n=5644). In the household contact dataset (n=1385) the point estimate for IGRA conversion at this cutoff (0⋅64, 0⋅51–0⋅81) was slightly higher than that for disease (0⋅79, 0⋅20–3⋅23; appendix pp 32–33; figure 3B).

Although the numbers of disease events in four of the five household contact studies were small (appendix p 34), resulting in wide confidence intervals, the observed protection against IGRA conversion was consistent across all five studies and statistically significant in two of these studies^24,26^ (appendix p 36).

In the ACSs, BCG vaccination showed no protection against sustained IGRA conversion at a cutoff of 0⋅7 IU/mL (aHR 0⋅90, 95% CI 0⋅81–1⋅02). There was indication of protection against tuberculosis disease among those with sustained conversion, although the confidence intervals crossed 1 (0⋅50, 0⋅24–1⋅04; appendix pp 29, 31; figure 3A). Among household contacts, there was no evidence of BCG vaccination protecting against sustained IGRA conversion at a cutoff of 0⋅7 IU/mL (0⋅54, 0⋅10–3⋅11).

In the ACSs, there was no association between BCG vaccination and sustained IGRA conversion at 0⋅35 IU/mL (aHR 1⋅01, 95% CI 0⋅93–1⋅11). There was indication of protection against tuberculosis disease among those with sustained conversion, but the 95% CIs crossed 1 (0⋅63, 0⋅36–1⋅11; appendix pp 32–33; figure 3B). Among household contacts, the small sample size precluded meaningful interpretation of the protective effect of BCG vaccination against sustained IGRA conversion at cutoff 0⋅35 IU/mL (0⋅56, 0⋅13–3⋅13).

Age-stratified models (two age strata for the ACSs and five age strata for the household contact studies and RCTs) showed similar patterns to the unstratified analyses though with wider confidence intervals, both for TST and IGRA conversion at the higher cutoffs (appendix pp 35, 37–41, 43). For both testing methods at the higher cutoff, in the ACSs among children aged 5–12 years, there was indication of protection against conversion but with 95% CIs that crossed 1 (TST 15 mm aHR 0⋅23, 95% CI 0⋅37–1⋅04; IGRA 0⋅7 IU/mL 0⋅51, 0⋅19–1⋅37).

Sex-stratified models showed similar patterns as the unstratified models for protection against TST conversion (appendix pp 42, 44–45, 47). At the 0⋅7 IU/mL cutoff, patterns for protection against IGRA conversion were also similar in the ACSs, and in the household contact studies the aHR differed between men (0⋅92, 95% CI 0⋅63–1⋅38) and women (0⋅52, 0⋅38–0⋅70; interaction term between sex and BCG vaccination p=0⋅024). aHRs for protection against IGRA conversion (0⋅52, 0⋅38–0⋅70) agreed with the aHRs for protection against disease (0⋅63, 0⋅12–3⋅36), although 95% CIs for the latter aHRs crossed 1; appendix pp 46, 48, 50).

When stratifying for latitude among household contacts, protection against IGRA conversion was most pronounced in studies done within the 20–40-degree band (aHR 0⋅63, 95% CI 0⋅53–0⋅86), and in the other bands numbers were insufficient for analysis (data not shown).

The reported sensitivity analyses focus on the IGRA findings. In the household contact studies, the observed protection against conversion was independent of the definition of IGRA conversion by different segments (appendix pp 4–7, 51; segment 2 aHR 0⋅65, 95% CI 0⋅50–0⋅84; segment 3 0⋅56, 0⋅31–1⋅10). Few or no tuberculosis disease events were observed in these segments. In the ACSs, where no protection for any definition of IGRA conversion was observed, the results remained unchanged.

When combining the analyses of the ACSs and household contacts in a single dataset, there was no association between BCG vaccination and IGRA (aHR 0⋅96, 95% CI 0⋅89–1⋅04), and sustained IGRA conversion (aHR 0⋅90, 0⋅81–1⋅00). Evidence of protection against disease was limited, both overall and among the infected measured by either single or sustained IGRA conversion (appendix p 53).

When limiting the IGRA analyses to participants for whom BCG vaccination status was ascertained from documentation rather than a scar, the observed pattern remained unchanged in the ACSs. In the three household contact studies where this information was available, the protective effect of BCG vaccination against conversion disappeared. Most of these datasets had only 15–30% unvaccinated participants, which precluded comparison between studies with high and low BCG vaccination coverage (data not shown).

When including baseline IGRA positive individuals in the household contact analysis, protection against IGRA positivity was still seen although was less pronounced (aHR 0⋅84, 95% CI 0⋅76–0⋅93). In this study sample population, we saw no protection against disease. Conversely, when we excluded baseline IGRA positive individuals from the ACS dataset, this resulted in BCG vaccination increasing the risk of IGRA conversion (1⋅20, 1⋅02–1⋅42; data not shown).

Additional sensitivity analyses (appendix pp 4–7) that considered no imputation of positive TST or IGRA before a positive tuberculosis test, including tuberculosis infection testing within 2 years post-vaccination, using Poisson instead of Cox proportional hazards for the ACSs, and accounting for BCG vaccination coverage, yielded no changes in results.

## Discussion

Our IPD meta-analysis of household contact studies found a protective effect of primary BCG vaccination against IGRA positivity measured by QuantiFERON at the cutoff of 0⋅7 IU/mL that was consistent with the observed protection against tuberculosis disease. The level of protection against QuantiFERON at the cutoff of 0⋅35 IU/mL was somewhat larger than the observed protection against disease, and no significant protection was observed against sustained IGRA conversion. These findings were not replicated in the ACS for which IGRA testing results were available. Our IPD meta-analysis of BCG vaccination trials, household contact studies, and ACSs showed no protection of primary BCG vaccination against TST conversion irrespective of the cutoff used.

We found that prevention of IGRA conversion is consistent with prevention of disease across household contact datasets and age groups. Importantly, this relationship remains when considering different proportions of the cohort that received BCG vaccination, thus mitigating concerns of selection bias because not all BCG vaccinations result in a typical scar.^22,24,25,34–36^ Contrary to findings in the household contact studies, BCG vaccination status was not associated with IGRA conversion in the ACSs. We found no explanation for this difference; however, it could have arisen from differences in study design, despite sensitivity analyses. First, household contacts were selected because of their recent household exposure, whereas the ACSs specifically focused on adolescents from high incidence settings. Second, five of the household contact studies had a follow-up time of 12 months, which might not have been long enough to observe all disease cases, because at least 40% of tuberculosis disease occurs more than a year after infection.^37^ Third, whereas in household contact studies *M tuberculosis* exposure predominantly occurred shortly before enrolment, it probably occurred more evenly over time in the ACSs, which might have biased our time-to-event analyses. However, incident rate analysis of the ACS data yielded similar results. We investigated the effect of including or excluding participants with positive IGRA at cohort baseline for various study types, but these variations were inconclusive. Although regional factors and context-specific variables in the South African ACSs might have played a role, we observed no such differences between the household contact datasets from South Africa versus those from other countries. Results from a larger set of household contacts suggest that BCG vaccination is ineffective in adolescents, which might have influenced the outcomes of the ACS studies, given their enrichment for this population.^3^

Our results suggest that TST-based prevention of *M tuberculosis* infection does not align with prevention of disease. This was corroborated by multiple sensitivity analyses. It is probable that vaccine-induced delayed-type hypersensitivity or boosting of TST reactivity by purified protein derivative in individuals before BCG vaccination or non-tuberculosis mycobacterial infection has influenced these results.^21–25^ Indeed, in the RCTs BCG vaccination was associated with a positive TST, which is likely to be BCG vaccination induced positivity,^26^ especially when a low cutoff for TST conversion was used. Our choice of a high cutoff for TST was aimed at minimising cross-reactivity from non-tuberculous mycobacteria and the BCG vaccine, thus enhancing specificity for detecting actual *M tuberculosis* infection. However, it is possible that this cutoff was still too low to exclude cross-reactions and boosting, but an even higher cutoff might lead to other problems such as false negativity.

Several limitations should be considered when interpreting our results. The diversity of cohorts and duration of follow-up might have impacted the observed outcomes. Most of the included studies were observational, which are more prone to selection bias as the reason for not having received the BCG vaccine might have been associated with the risk of *M tuberculosis* infection or tuberculosis disease. Additionally, most of these studies were not initially intended for this specific research question. Despite our attempts to control confounding through multivariable analysis, unmeasured variables, such as socioeconomic status, might have influenced the results. The variety of tests used to ascertain disease endpoints across the included studies might have introduced misclassification bias. In our included studies, the majority of tuberculosis disease ascertainment was based on tuberculosis-suggestive symptoms without specifying the symptoms. As a result, our findings cannot be extrapolated to subclinical tuberculosis. Because of a small number of household contacts with sustained IGRA conversion, we adjusted the definition to a 3-month period; this adjustment did not yield different results. Contrary to our expectation of observing an increased number of positive TST results within the first 2 years following BCG vaccination, driven by increased TST cross-reactivity during this period, our analysis revealed no substantial differences in outcomes. This finding could be attributed to the high cutoff we employed, aimed at minimising false positive results. Alternatively, the impact of BCG vaccination on TST outcomes might be less pronounced than initially expected.^7,8,11^

Our study employed IGRA and TST tests as measurement of *M tuberculosis* infection. Previous research cautioned against using BCG-related tuberculin conversion as a reliable measure of the immunological impact of the BCG vaccine, thus precluding its consideration as a correlate of protection in this analysis.^38,39^ TST responses to BCG vaccination are noted to be variable and challenging to interpret, especially in regions with high tuberculosis endemicity.

Finally, a formal surrogate endpoint analysis was considered but deemed unsuitable as we do not consider prevention of *M tuberculosis* infection a surrogate endpoint to be used for licensure and because of the small number of disease endpoints in the IPD meta-analysis that also hindered interpretation of protection for prevention of disease estimates.

Despite these limitations, our study offers insights for the potential of *M tuberculosis* infection tests as a proxy for prevention of disease following BCG vaccination, across various definitions. We explored the protective effect of BCG vaccination among diverse study cohorts and provided a comprehensive evaluation of the vaccine’s performance in different demographic groups. The current pipeline holds two other mycobacterial live-attenuated tuberculosis vaccines. Our study could inform on assessing the effectiveness of these tuberculosis vaccines against infection among different groups. Positive IGRA could be a surrogate for other live-attenuated and potentially non-bacterial vaccines, but trials would need to be carried out to measure its predictive ability. As new tuberculosis biomarkers emerge, future trials could explore combining these with QFT to potentially enhance predictive capacity for tuberculosis progression.^40^ Understanding these complex relationships is of vital importance for informing tuberculosis prevention strategies and optimising vaccine development efforts.

BCG vaccination protection against infection measured as QuantiFERON conversion exhibits inconsistent results across different groups. Among groups with recent household exposure, QuantiFERON conversion was consistent with protection against disease and could be evaluated as a proxy for disease in tuberculosis vaccine trials, pending additional research and validation. We found that TST lacks value for prevention of disease in phase 2b proof-of-concept trials.

## Supplementary Material

references

appendix

reference

## Figures and Tables

**Table:** Study characteristics of datasets included in individual patient data meta-analysis.

	Country	Study type	Number of participants	Study population	Start year	Duration of follow-up	BCG vaccination scar, vaccination card, or scar and vaccination card	Infection tests*	Bacteriologically confirmed tuberculosis

Mumpe-Mwanja et al (2015)^17^	Uganda	Prospective cohort	4981	Adolescent cohort	2009	24 months	Scar	Three TST	Yes
Mahomed et al (2013)^18^	South Africa	Prospective cohort	6363	Adolescent cohort	2005	24–34 months	Scar and vaccination card	Two TST or two IGRA; or five TST or eight IGRA	Yes
Carvalho et al (2001)^22^	Brazil	Prospective cohort	360	Household contacts	1995	12 months	Scar	Four TST	Not specified
Aibana et al (2016)^23^	Peru	Prospective cohort	12 648	Household contacts	2009	12 months	Scar	Three TST	Yes
Acuña-Villaorduña et al (2018)^24^	Brazil	Prospective cohort	894	Household contacts	2008	21 months	Scar	Two TST or one IGRA	Yes
Verhagen et al (2014)^25^	Venezuela	Prospective cohort	163	Household contacts aged <16 years	2010	12 months	Scar and vaccination card	Three TST or two IGRA	Yes
del Corral et al (2009)^26^	Colombia	Prospective cohort	2060	Household contacts	2005	24–36 months	Scar and vaccination card	Two TST or two IGRA	Yes
Espinal et al (2000)^27^	Dominican Republic	Prospective cohort	803	Household contacts	1994	14 months	Scar	Three TST	Both probable and bacteriologically confirmed tuberculosis
Huerga et al (2019)^28^	Armenia	Prospective cohort	150	Household child contacts	2012	24 months	Scar and vaccination card	Eight TST or five IGRA	Yes
Chan et al (2014)^29^	Taiwan	Retrospective cohort	9411	Household child contacts	2008–09	22–46 months	Vaccination card	Two TST	Both probable and bacteriologically confirmed tuberculosis
Jones-López et al (2013)^32^	Uganda	Retrospective cohort	442	Household contacts	2009	47 months	Scar	Two TST or two IGRA	Not specified
Lemos et al (2004)^30^	Brazil	Prospective cohort	272	Household contacts	1997	12 months	Scar	Three TST	Yes
Mandalakas et al (2021)^31^	South Africa	Prospective cohort	1343	Child contacts	2008	15 months	Scar and vaccination card	Three TST or three IGRA	Yes
Aronson et al (2004)^33^	USA	Clinical trial	2963	Child and adolescent population	1935	0–15 years and 15–55 years^†^	Randomised to receive vaccine^‡^	15 TST	Both probable and bacteriologically confirmed tuberculosis

BCG=Bacillus Calmette-Guérin. IGRA=IFN-γ release assay. TST=tuberculin skin test.

* Infection measurement indicates approximate number of tests conducted throughout the study period.

† Initial active follow-up conducted for 15 years, after this passive follow-up through reporting.

‡ BCG-Phipps strain 317 (Pasteur Institute, Paris, France) in 1926; strain 575 (Pasteur Institute, Paris, France) in 1938.

## Data Availability

The individual participantdata cannotbe made available to othersbecause of restrictions given to the authors from the data owners and participants. The study protocol can be made available upon reasonable request to the corresponding author.

## Research in context

### Evidence before this study

This individual patient data meta-analysis was built upon the foundation of four systematic reviews. We used one review from 2013, another from 2014, and two reviews from 2020. These systematic reviews form the basis for our current IPD analysis, providing a comprehensive overview of the available data spanning from the 1900s to 2019.

There are challenges associated with tuberculosis vaccine trials using disease as an outcome due to their sizeable scale, high cost, and long duration. Alternative predictors of tuberculosis disease prevention would facilitate vaccine development. Prevention of *Mycobacterium tuberculosis* infection has been proposed as an endpoint for phase 2b proof-of-concept vaccine trials. By quantitatively assessing the consistency between the preventive effectiveness against *M tuberculosis* infection and the subsequent prevention of disease effect, prevention of *M tuberculosis* infection might be considered for phase 2b proof-of-concept trials of the Bacillus Calmette-Guérin (BCG) vaccine or vaccines with similar mode of action.

No previous research has systematically addressed this study question using individual participant data. Several systematic reviews have investigated infection and disease prevention from BCG vaccination separately or the predictive value of tests without incorporating vaccination.

### Added value of this study

This study reviewed individual patient data from randomised controlled trials (RCTs), cohort studies, and household contact studies on the consistency between the preventive efficacy against *M tuberculosis* infection and the preventive effectiveness against disease, taking into consideration different infection tests and cutoff values. We evaluated whether the effectiveness of BCG vaccination for prevention of *M tuberculosis* infection was consistent with that for prevention of tuberculosis disease. Six cohort studies (five household contact) and none of the RCTs had IFN-γ release assay (IGRA) data. 13 cohort studies (11 household contact) and one RCT had tuberculin skin test (TST) data. The follow-up of the studies ranged between 1 year and 55 years.

### Implications of all the available evidence

A TST-based *M tuberculosis* infection endpoint provided no information about protection against disease, arguing against the value of using TST as a measure of infection. Although the definition (single or sustained conversion) and cutoff value for a positive test needs to be considered, BCG vaccine protection against infection measured as QuantiFERON conversion in household contact studies was consistent with protection against disease. Further research might explore its use as a surrogate endpoint in future vaccine studies. Because vaccine trials with IGRA-based endpoints can be considerably smaller in size and shorter in duration than trials with disease endpoints, selecting vaccine candidates based on phase 2b trials with IGRA-based endpoints potentially offers a more efficient approach, shortening timelines and resource requirements. Whether IGRA-based assays apply to other vaccines beyond BCG and similar live-attenuated vaccine candidates remains to be seen, which underscores the importance of scrutinising available data to optimise trial methodologies and accelerate scientific advancements in the field.
