# Supplementary material for: Effectiveness of the primary Bacillus Calmette-Guérin vaccine against the risk of Mycobacterium tuberculosis infection and tuberculosis disease: a meta-analysis of individual participant data
Source: Lancet Microbe. Author manuscript; Available in PMC 2026 Jan 7. (PMC12778190; doi:10.1016/j.lanmic.2024.100961)
Supplement: references [file NIHMS2101069-supplement-references.pdf]

References for BCG Vaccine Meta-Analysis Article

APA (7th)

Pelzer, P. T., Stuck, L., Martinez, L., Richards, A. S., Acuña-Villaorduña, C., Aronson, N. E., … Cobelens, F. G. J. (2025). Effectiveness of the primary Bacillus Calmette-Guérin vaccine against the risk of Mycobacterium tuberculosis infection and tuberculosis disease: A meta-analysis of individual participant data. The Lancet Microbe, 6(2), 100961. https://doi.org/10.1016/j.lanmic.2024.100961

AMA

Pelzer PT, Stuck L, Martinez L, Richards AS, Acuña-Villaorduña C, Aronson NE, et al. Effectiveness of the primary Bacillus Calmette-Guérin vaccine against the risk of Mycobacterium tuberculosis infection and tuberculosis disease: a meta-analysis of individual participant data. Lancet Microbe. 2025;6(2):100961. doi:10.1016/j.lanmic.2024.100961

Vancouver

Pelzer PT, Stuck L, Martinez L, Richards AS, Acuña-Villaorduña C, Aronson NE, et al. Effectiveness of the primary Bacillus Calmette-Guérin vaccine against the risk of Mycobacterium tuberculosis infection and tuberculosis disease: a meta-analysis of individual participant data. Lancet Microbe. 2025;6(2):100961. doi:10.1016/j.lanmic.2024.100961. PMID: 39709975.

Chicago

Pelzer, Puck T., Logan Stuck, Leonardo Martinez, Alexandra S. Richards, Carlos Acuña-Villaorduña, Naomi E. Aronson, et al. "Effectiveness of the Primary Bacillus Calmette-Guérin Vaccine against the Risk of Mycobacterium tuberculosis Infection and Tuberculosis Disease: A Meta-Analysis of Individual Participant Data." The Lancet Microbe 6, no. 2 (2025): 100961. https://doi.org/10.1016/j.lanmic.2024.100961.

MLA (9th)

Pelzer, Puck T., et al. "Effectiveness of the Primary Bacillus Calmette-Guérin Vaccine against the Risk of Mycobacterium tuberculosis Infection and Tuberculosis Disease: A Meta-Analysis of Individual Participant Data." The Lancet Microbe, vol. 6, no. 2, 2025, p. 100961. https://doi.org/10.1016/j.lanmic.2024.100961.

BibTeX

```
@article{Pelzer2025BCG_IPD,
  title   = {Effectiveness of the primary Bacillus Calmette-Guérin vaccine against the risk of Mycobacterium tuberculosis infection and tuberculosis disease: a meta-analysis of individual participant data},
  author  = {Pelzer, Puck T. and Stuck, Logan and Martinez, Leonardo and Richards,
```

```
Alexandra S. and Acu{\~n}a-Villaordu{\~n}a, Carlos and Aronson, Naomi E. and et al.},
  journal = {The Lancet Microbe},
  year    = {2025},
  volume  = {6},
  number  = {2},
  pages   = {100961},
  doi     = {10.1016/j.lanmic.2024.100961},
  pmid    = {39709975}
}
```
