# Supplementary material for: Effectiveness of the primary Bacillus Calmette-Guérin vaccine against the risk of Mycobacterium tuberculosis infection and tuberculosis disease: a meta-analysis of individual participant data
Source: Lancet Microbe. Author manuscript; Available in PMC 2026 Jan 7. (PMC12778190; doi:10.1016/j.lanmic.2024.100961)
Supplement: appendix [file NIHMS2101069-supplement-appendix.pdf]

References: Effectiveness of the primary Bacillus Calmette-Guérin vaccine against the risk of Mycobacterium tuberculosis infection and tuberculosis disease (Meta-analysis)

Abubakar I, Pimpin L, Ariti C, et al. Systematic review and meta-analysis of the current evidence on the duration of protection by Bacillus Calmette-Guérin vaccination against tuberculosis. Health Technol Assess. 2013;17(37):1–372.

Andersen P, Doherty TM. The success and failure of BCG — implications for a novel tuberculosis vaccine. Nat Rev Microbiol. 2005;3(8):656–62.

Colditz GA, Brewer TF, Berkey CS, et al. Efficacy of BCG vaccine in the prevention of tuberculosis: meta-analysis of the published literature. JAMA. 1994;271(9):698–702.

Fine PEM. Variation in protection by BCG: implications of and for heterologous immunity. Lancet. 1995;346(8986):1339–45.

Mangtani P, Abubakar I, Ariti C, et al. Protection by BCG vaccine against tuberculosis: a systematic review of randomized controlled trials. Clin Infect Dis. 2014;58(4):470–80.

Nemes E, Geldenhuys H, Rozot V, et al. Prevention of M. tuberculosis infection with H4:IC31 vaccine or BCG revaccination. N Engl J Med. 2018;379:138–49.

Roy A, Eisenhut M, Harris RJ, et al. Effect of BCG vaccination against Mycobacterium tuberculosis infection in children: systematic review and meta-analysis. BMJ. 2014;349:g4643.

Trunz BB, Fine P, Dye C. Effect of BCG vaccination on childhood tuberculous meningitis and miliary tuberculosis worldwide: a meta-analysis and assessment of cost-effectiveness. Lancet. 2006;367(9517):1173–80.

World Health Organization. BCG vaccines: WHO position paper – February 2018. Wkly Epidemiol Rec. 2018;93(8):73–96.
