# Supplementary material for: Effectiveness of the primary Bacillus Calmette-Guérin vaccine against the risk of Mycobacterium tuberculosis infection and tuberculosis disease: a meta-analysis of individual participant data
Source: Lancet Microbe. Author manuscript; Available in PMC 2026 Jan 7. (PMC12778190; doi:10.1016/j.lanmic.2024.100961)
Supplement: reference [file NIHMS2101069-supplement-reference.pdf]

References

Abubakar, I., Pimpin, L., Ariti, C., Beynon, R., Mangtani, P., Sterne, J. A., ... & Rodrigues, L. C. (2013). Systematic review and meta-analysis of the current evidence on the duration of protection by bacillus Calmette-Guérin vaccination against tuberculosis. Health Technology Assessment, 17(37), 1–372.

Colditz, G. A., Brewer, T. F., Berkey, C. S., Wilson, M. E., Burdick, E., Fineberg, H. V., & Mosteller, F. (1994). Efficacy of BCG vaccine in the prevention of tuberculosis: meta-analysis of the published literature. JAMA, 271(9), 698–702.

Mangtani, P., Abubakar, I., Ariti, C., Beynon, R., Pimpin, L., Fine, P. E., ... & Rodrigues, L. C. (2014). Protection by BCG vaccine against tuberculosis: a systematic review of randomized controlled trials. Clinical Infectious Diseases, 58(4), 470–480.

Nemes, E., Geldenhuys, H., Rozot, V., Rutkowski, K. T., Ratangee, F., Bilek, N., ... & Hawn, T. R. (2018). Prevention of M. tuberculosis infection with H4:IC31 vaccine or BCG revaccination. New England Journal of Medicine, 379(2), 138–149.

Roy, A., Eisenhut, M., Harris, R. J., Rodrigues, L. C., Sridhar, S., Habermann, S., ... & Abubakar, I. (2014). Effect of BCG vaccination against Mycobacterium tuberculosis infection in children: systematic review and meta-analysis. BMJ, 349, g4643.

Trunz, B. B., Fine, P., & Dye, C. (2006). Effect of BCG vaccination on childhood tuberculous meningitis and miliary tuberculosis worldwide: a meta-analysis and assessment of cost-effectiveness. The Lancet, 367(9517), 1173–1180.

World Health Organization. (2018). BCG vaccines: WHO position paper – February 2018. Weekly Epidemiological Record, 93(8), 73–96.
